# Hybrid Optimization Path Planning Method for AGV Based on KGWO

**DOI:** 10.3390/s24185898

**Published:** 2024-09-11

**Authors:** Zhengjiang Guo, Yingkai Xia, Jiawei Li, Jiajun Liu, Kan Xu

**Affiliations:** 1 College of Engineering, Huazhong Agricultural University, Wuhan 430070, China; 2 Wuhan Second Ship Design and Research Institute, Wuhan 430205, China

**Keywords:** automated guided vehicles, path planning, grey wolf optimization, Kalman filter, partially matched crossover

## Abstract

To address the path planning problem for automated guided vehicles (AGVs) in challenging and complex industrial environments, a hybrid optimization approach is proposed, integrating a Kalman filter with grey wolf optimization (GWO), as well as incorporating partially matched crossover (PMX) mutation operations and roulette wheel selection. Paths are first optimized using GWO, then refined with Kalman filter corrections every ten iterations. Moreover, roulette wheel selection guides robust parent path selection, while an elite strategy and partially matched crossover (PMX) with mutation generate diverse offspring. Extensive simulations and experiments were carried out under a densely packed goods scenario and complex indoor layout scenario, within a fully automated warehouse environment. The results showed that this hybrid method not only enhanced the various optimization metrics but also ensured more predictable and collision-free navigation paths, particularly in environments with complex obstacles. These improvements lead to increased operational efficiency and safety, highlighting the method’s potential in real-world applications.

## 1. Introduction

Automated guided vehicles (AGVs) [1] have become increasingly vital in modern logistics and industrial automation, due to their ability to enhance efficiency and reduce labor costs. AGVs are widely deployed in unmanned environments such as automated warehouses, ports, and factories, to perform tasks like material handling, transportation, and loading/unloading [2]. With the rapid advancement in automation technology and the increasing complexity of operational environments, the need for advanced path planning algorithms capable of optimizing AGV navigation in static and complex industrial environments has become critical.

The primary goal of this work was to develop a robust path planning algorithm that enhances AGV performance by optimizing key parameters such as path length, path smoothness, and computational efficiency in static and complex environments. Path planning [3] is a crucial aspect of mobile robot intelligence, enabling robots to autonomously navigate from start to goal positions. Depending on the scope, path planning can be divided into global path planning and local path planning. Global path planning aims to find the shortest path from start to end points on a known environment map, while avoiding collisions with obstacles [4]. Local path planning considers the robot’s motion model and can plan paths in completely unknown or partially known environments, effectively avoiding obstacles based on local environmental information. Since AGVs are predominantly used in static environments where obstacles are fixed or change slowly [5], which eliminates the need for dynamic obstacle avoidance, global path planning in challenging and complex industrial environments is considered in this study.

Traditional path planning methods include algorithms such as A* [6], Dijkstra [7], and Bellman–Ford. While these methods perform well in finding the shortest path, they exhibit low computational efficiency when dealing with large-scale complex environments. Moreover, these methods heavily rely on the environment, and any changes necessitate a complete recalculation of the path, making them ineffective in dynamic settings [8].

In recent decades, metaheuristic algorithms have been increasingly applied to path planning problems. These algorithms, including particle swarm optimization (PSO) [9], genetic algorithms (GA) [10], differential evolution (DE) [11], gradient-based optimization (GBO) [12], ant colony optimization (ACO) [13], cuckoo search (CS) [14], firefly algorithm (FA) [15], whale optimization algorithm (WOA) [16], ant lion optimizer (ALO) [17], virus colony search (VCS), slime mould algorithm (SMA), and hawk optimization algorithm (HHO) [18], offer significant advantages in solving complex path planning problems. They provide flexible and adaptive approaches capable of efficiently exploring large search spaces and avoiding local optima.

Despite their numerous advantages, many metaheuristic algorithms still face challenges, such as long convergence times, suboptimal paths, and local optima [19]. In this work, the grey wolf optimization (GWO) algorithm is utilized to optimize critical parameters such as path length and smoothness, with the goal of achieving faster convergence and improved path quality in static and complex environments. Compared to other metaheuristic algorithms, the grey wolf optimization (GWO) algorithm [20] offers several advantages, including its simplicity, having fewer parameters to adjust, and excellent balance of exploration and exploitation. Due to these strengths, GWO has garnered significant attention from researchers since its inception.

The grey wolf optimization (GWO) algorithm, inspired by the social hierarchy and hunting behavior of grey wolves, was introduced by [21] in 2014. It has proven effective in solving highly nonlinear, multivariate, and multimodal optimization problems. GWO mimics the leadership hierarchy and hunting mechanism of grey wolves in nature, involving four types of grey wolves: alpha, beta, delta, and omega, which guide the search process. This algorithm has shown great potential in various optimization tasks, due to its ability to balance exploration and exploitation phases effectively.

Several researchers have attempted to enhance GWO for path planning. For instance, Cheng et al. proposed an improved PSO integrated with GWO (IPSO-GWO) [21], introducing chaos and adaptive inertia weights to enhance the global search capabilities. However, they still faced challenges in parameter tuning and overfitting. Li et al. combined ACO with GWO, using GWO for the initial path search and integrating optimized solutions into the ACO pheromone model, improving the exploration capabilities but being susceptible to local optima. Zhang et al. proposed adaptive GWO (AGWO), which adjusts convergence factors adaptively and updates weight factors, demonstrating excellent performance in convergence accuracy and speed, but heavily relying on the accuracy of environmental models and real-time data [22].

To address these issues, this paper proposes a novel method called Kalman filter–grey wolf optimization (KGWO) for AGV path planning. The KGWO algorithm is designed to optimize path length, smoothness, and computational efficiency by leveraging the global search capabilities of GWO and the local optimization benefits of a Kalman filter [10,23]. Additionally, it incorporates an elite strategy and partially matched crossover (PMX) [19,24] mutation operations to enhance the overall optimization performance. By integrating these techniques, the KGWO algorithm aims to reduce convergence time, improve path smoothness, and optimize path length. The elite strategy ensures that the best solutions are retained and propagated through the generations, while the PMX mutation introduces diversity and helps escape local optima [25,26]. This hybrid approach not only accelerates the convergence process but also increases the robustness of the algorithm, making it less susceptible to getting stuck in local optima. Consequently, the KGWO algorithm ensures efficient and reliable AGV navigation, even in complex and cluttered environments [27,28]. The specific parameters optimized by the GWO in this study include path length, smoothness, and the balance between exploration and exploitation, to achieve efficient and effective AGV path planning.

To the best of the authors’ knowledge, no prior work has explored the integration of GWO with a Kalman filter and PMX mutation for enhancing AGV path planning in complex environments. The main contributions of this work can be summarized as follows:

1. The integration of GWO and a Kalman filter enhances AGV path planning in static and complex environments. GWO’s global search capabilities combined with the Kalman filter’s local optimization reduce the convergence time and improve path smoothness and length [29]. This hybrid approach efficiently explores the solution space and refines solutions, leading to more accurate and effective path planning, especially in complex terrains where traditional methods may falter [30].
2. The use of an elite strategy and PMX mutation operations enhances the optimization performance. The elite strategy retains and propagates the best solutions, accelerating convergence. PMX mutation introduces diversity, preventing premature convergence and aiding in escaping local optima. This dual approach makes the algorithm more robust and adaptable, ensuring consistent performance across various scenarios.
3. Extensive simulation and experimental results under two automated warehouse scenarios demonstrated the superior performance of the KGWO. In the packed storage setup, the KGWO algorithm efficiently navigated narrow aisles and closely spaced obstacles, optimizing the path length and smoothness. In the complex indoor layout, it handled intricate pathways and static obstacles, ensuring a more predictable and collision-free navigation. These improvements in speed and path length compared to conventional methods highlighted the KGWO algorithm’s capability to provide reliable and efficient AGV path planning, enhancing overall operational efficiency in static and complex environments.

The remainder of this paper is organized as follows: Section 2 introduces the environment and path planning mission. Section 3 delves into a detailed explanation of the proposed KGWO algorithm. Section 4 verifies the superiority of the proposed algorithm through MATLAB simulation under different map environments. Section 5 demonstrates the results of real experiments conducted using an AGV. Section 6 concludes the paper by summarizing the main findings.

## 2. Environment Introduction and Path Planning Mission

**Figure 1:**
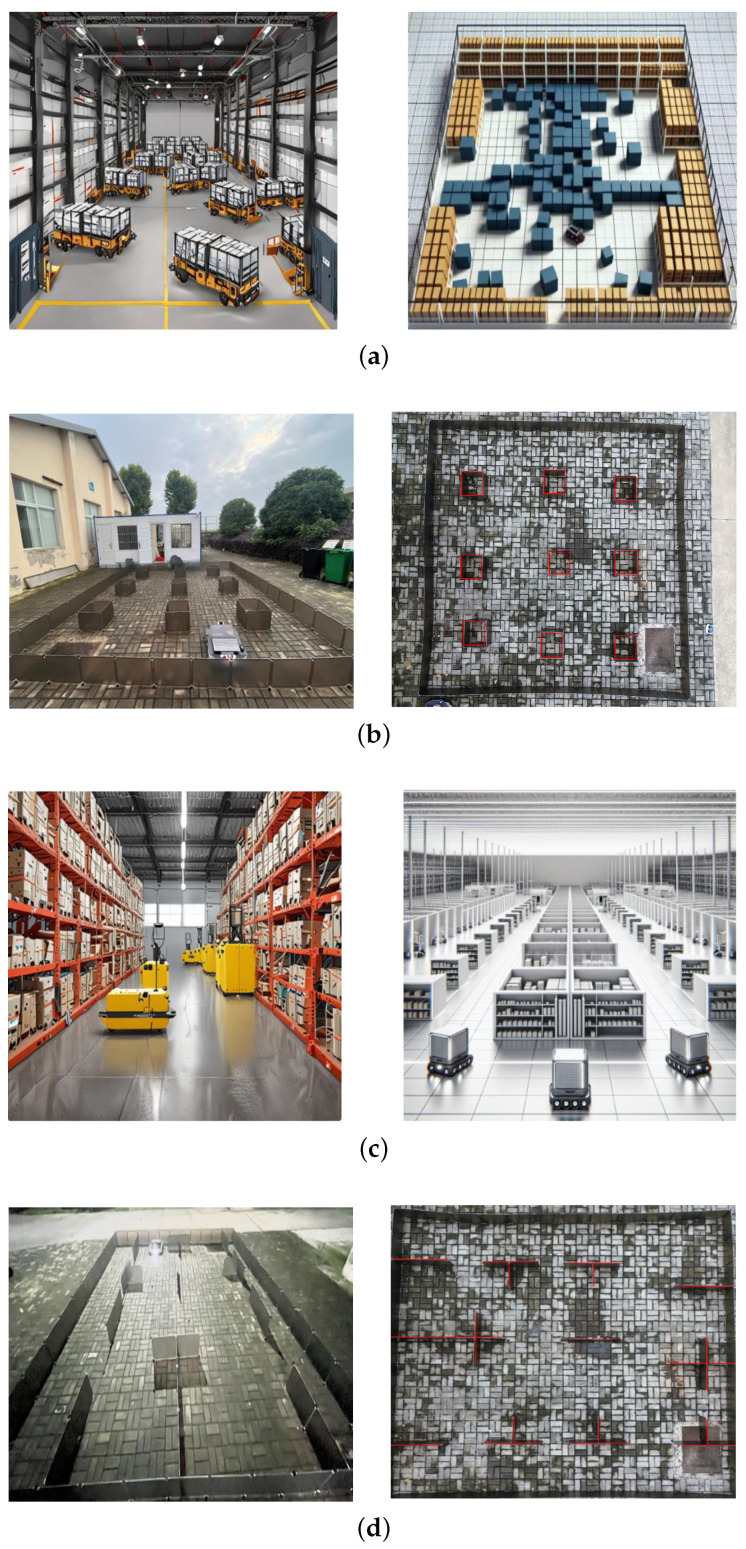
Various scenarios and their environment constructions. (**a**) Densely packed goods scenario. (**b**) Environment construction for densely packed goods scenario. (**c**) Complex indoor layout scenario. (**d**) Environment construction for complex indoor layout scenario.

The unified Fully Automated Warehouse Environment [31] (see Figure 1a,c) was chosen to simulate real-world AGV operational scenarios, ensuring a comprehensive evaluation of the proposed algorithm’s performance under varied and challenging conditions. This environment replicates the complexity and diversity of actual warehouse and industrial settings, allowing for a thorough assessment of the algorithm’s robustness and adaptability. By incorporating distinct working conditions, the testing framework was designed to highlight the strengths and potential limitations of the AGV path planning method [31,32]. This approach ensured that the algorithm could effectively handle different types of obstacles and spatial constraints, providing valuable insights into its practical applicability. The detailed evaluation in this environment confirmed the algorithm’s capability to enhance operational efficiency and safety in automated warehouse systems.

Densely packed goods scenario: Nine square boxes are arranged in a 3 × 3 grid at the center, serving as obstacles (see Figure 1b), to simulate a typical dense warehouse environment, where goods are densely packed and AGVs must navigate efficiently through narrow passages. Path planning in this scenario is critical for finding the shortest collision-free path, which is essential for operational efficiency and safety. Experiments in this environment allowed for the evaluation of the algorithm’s performance in handling complex path planning tasks, ensuring it could navigate the shortest path, while avoiding collisions.

Complex indoor layout scenario: A symmetrical layout was constructed with partitions and barriers, resembling room structures (see Figure 1d), to simulate environments such as factories or office buildings, where AGVs operate in more complex and constrained spaces. The partitions and barriers represented walls and fixed equipment, requiring the AGVs to navigate through more intricate and narrow spaces. This environment tested the algorithm’s efficiency in navigating through multiple compartments and avoiding obstacles, emphasizing the importance of obstacle avoidance and path optimization in complex indoor environments.

## 3. Dynamic Modeling and Control Strategies for AGV Systems

### 3.1. AGV System Dynamics

Before delving into the improvements made to the grey wolf optimization (GWO) algorithm, it is essential to discuss the system dynamics that governed the automated guided vehicle (AGV) in the industrial environment. The system dynamics model plays a crucial role in ensuring that the planned paths are not only theoretically optimal but also practically feasible for the AGV to follow, given its physical and dynamic constraints.

**Figure 2:**
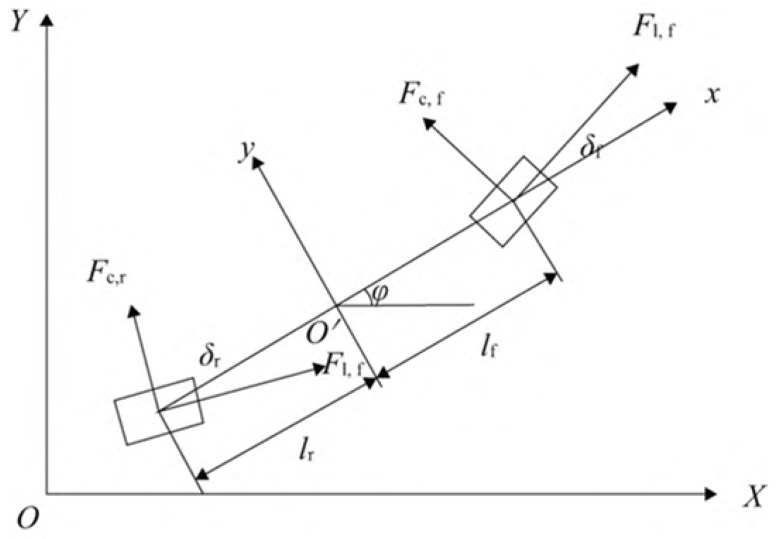
The two-degree-of-freedom dynamic model of the AGV.

The AGV studied in this work adopts an eight-wheel four-drive structure, with independent drive steering for the front and rear wheels. Four universal wheels are symmetrically distributed along the vehicle’s central axis to provide support and balance. Based on this platform, to improve the real-time performance and stability of the control algorithm, the vehicle model was appropriately simplified on the basis of accurately describing the vehicle dynamics process. Thus, a two-degree-of-freedom dynamic model was established, as shown in Figure 2.

Here, XOY is the global coordinate system referenced to the Earth coordinate system, and $xO^{\prime }y$ is the vehicle coordinate system referenced to the AGV’s own axis. The origin of the vehicle coordinate system is at the AGV’s center of mass, with the *x*-axis along the vehicle’s axis and the *y*-axis perpendicular to the *x*-axis [33]. $v_{x}$ and $v_{y}$ represent the longitudinal and lateral velocities at the center of mass in the vehicle coordinate system, ϕ is the yaw angle, *m* is the vehicle mass, and $I_{z}$ is the moment of inertia about the *z*-axis.

The vehicle dynamics model was established based on Newton’s second law:

(1) $$\begin{array}{cc} m{\dot{v}}_{x} & =m\dot{\phi }+2\left(F_{l,f}\cos\delta_{f}-F_{c,f}\sin\delta_{f}\right)+2\left(F_{l,r}\cos\delta_{r}-F_{c,r}\sin\delta_{r}\right)\\ m{\dot{v}}_{y} & =-m\dot{\phi }+2\left(F_{l,f}\cos\delta_{f}+F_{c,f}\sin\delta_{f}\right)+2\left(F_{l,r}\cos\delta_{r}+F_{c,r}\sin\delta_{r}\right)\\ I_{z}\dot{\phi } & =2l_{f}\left(F_{l,f}\cos\delta_{f}+F_{c,f}\sin\delta_{f}\right)-2l_{r}\left(F_{l,r}\cos\delta_{r}+F_{c,r}\sin\delta_{r}\right)\\ \dot{X} & =\dot{x}\cos\phi -\dot{y}\sin\phi \\ \dot{Y} & =\dot{x}\sin\phi +\dot{y}\cos\phi \end{array}$$

Under the small-angle assumption, the trigonometric functions satisfy cosθ≈1, sinθ≈θ, tanθ≈θ. Under this assumption, the tire slip angles for the front and rear wheels can be expressed as

(2) $$\begin{array}{cc} \alpha_{f} & =\delta_{f}-\frac{\dot{y}+l_{f}\dot{\phi }}{v_{x}}\\ \alpha_{r} & =\frac{l_{r}\dot{\phi }-\dot{y}}{v_{x}} \end{array}$$

Assuming a low-slip condition, the lateral and longitudinal forces of the tires can be expressed in the following linear form:

(3) $$\begin{array}{cc} F_{c,f} & =C_{c,f}\alpha_{f},\;F_{c,r}=C_{c,r}\alpha_{r}\\ F_{l,f} & =C_{l,f}s_{f},\;F_{l,r}=C_{l,r}s_{r} \end{array}$$

where $C_{c,f}$ and $C_{c,r}$ are the cornering stiffness coefficients for the front and rear tires, respectively; $\alpha_{f}$ and $\alpha_{r}$ are the slip angles for the front and rear tires, respectively; $C_{l,f}$ and $C_{l,r}$ are the longitudinal stiffness coefficients for the front and rear tires, respectively; and $s_{f}$ and $s_{r}$ are the slip ratios for the front and rear tires, respectively.

These dynamic equations can be expressed in the form of a state-space equation:

(4) $$\dot{\xi }=f\left(\xi ,u\right)$$

where the system state vector is $\xi ={\left[\dot{x},\dot{y},\phi ,\dot{\phi },X,Y\right]}^{T}$, and the control input is $u=\delta_{f}$.

By establishing a comprehensive system dynamics model, the proposed path planning algorithm could better navigate complex industrial environments [34], addressing both the physical and operational constraints of the AGV. This integration laid the foundation for the improvements discussed in the subsequent section on the grey wolf optimization algorithm.

### 3.2. Control Strategies for AGV Systems

The successful operation of an AGV system relies not only on the optimal paths generated by algorithms like grey wolf optimization (GWO) but also on effective control methods that can execute these paths in real-world environments. One of the most commonly used control methods in AGV systems is the proportional-integral-derivative (PID) controller.

A PID controller continuously calculates [33] an error value as the difference between a desired setpoint (e.g., the target trajectory) and the actual position or velocity of the AGV. The controller then applies corrections based on three terms:

(5) $$u\left(t\right)=K_{p}e\left(t\right)+K_{i}\int e\left(t\right)\;dt+K_{d}\frac{de(t)}{dt}$$

where u(t) is the control output, e(t) is the error, $K_{p}$ is the proportional gain, $K_{i}$ is the integral gain, and $K_{d}$ is the derivative gain. The combined output from these three terms is used to adjust the AGV’s steering angle, speed, and other relevant control parameters, ensuring smooth and accurate navigation.

In an industrial setting, the AGV may encounter obstacles or variations in surface conditions that cause deviations from its planned trajectory. The PID controller mitigates these deviations by dynamically adjusting the control inputs, ensuring that the AGV remains on its intended path. The optimal parameters for the PID controller, such as the gains for the proportional, integral, and derivative terms, can be fine-tuned using the GWO algorithm, allowing the AGV to achieve both stability and responsiveness in its movements.

## 4. Improved Grey Wolf Optimization Algorithm Based on a Kalman Filter

### 4.1. Grey Wolf Optimization Algorithm

**Figure 3:**
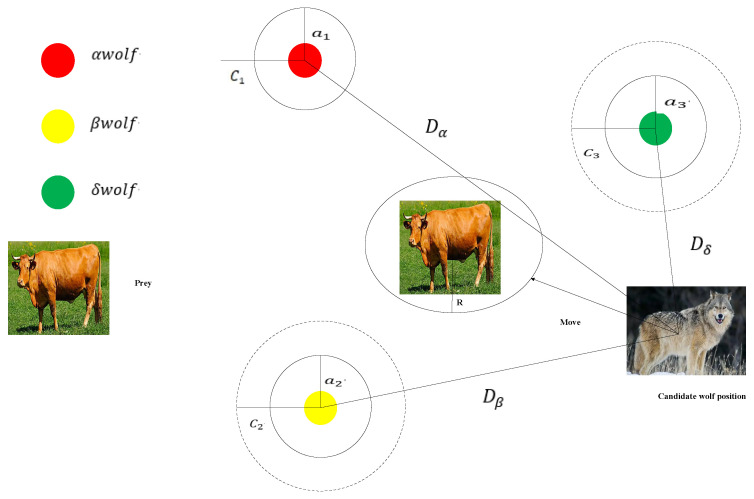
GWO algorithm principle diagram.

The grey wolf optimizer (GWO) algorithm is inspired by the natural behavior, internal mechanisms, and hunting strategies of grey wolves, establishing a hierarchical task system. Based on a hierarchy, the grey wolf population is divided into four categories: alpha (α), beta (β), delta (δ), and omega (ω). As shown in Figure 3, the social hierarchy of grey wolves is distinct. In this optimization algorithm, the results correspond to these four types of individuals. The position of the α wolves represents the best solution found so far. The position of the β wolves represents the second-best solution, while the δ wolves’ position is the third-best solution. The remaining candidate solutions are provided by the ω wolves. When the grey wolf pack hunts, their main behaviors include encircling prey, hunting, and finally attacking the prey.

The encircling behavior can be mathematically modeled using the following equations:

(6) $$D=\left|C*X_{P}\left(t\right)-X\left(t\right)\right|$$

(7) $$X_{i}\left(t+1\right)=X_{P}\left(t\right)-A*D$$

where *D* represents the distance between the individual and the target; *t* is the current iteration number; and $X_{P}\left(t\right)$ is the prey’s current location coordinate. *A* and *C* are coefficient vectors calculated as follows:

(8) $$A=2a*r_{1}-a$$

(9) $$C=2a*r_{2}$$

(10) $$a=2-\frac{2t}{t_{\max}}$$

In these equations, *a* is a convergence factor that decreases linearly from 2 to 0 over the course of the iterations. $r_{1}$ and $r_{2}$ are random vectors in the range [0, 1].

Grey wolves have the ability to recognize the position of prey and encircle them. In optimization problems, grey wolves are considered capable of identifying the potential location of the prey (optimal solution). Once the position of the prey has been identified, the α,β, and δ wolves guide the pack to encircle it. In the decision space of optimization problems, the exact location of the best solution (the prey’s position) is unknown. Therefore, to simulate the hunting behavior, it is assumed that the α,β, and δ wolves have a better ability to recognize potential prey positions. In each iteration, the best three wolves (α,β,δ) are kept, and the positions of other search agents (including ω wolves) are updated based on their position. The mathematical model for grey wolves tracking prey is described as follows:

(11) $$D_{\alpha }=\left|C_{1}*X_{\alpha }-X\right|$$

(12) $$D_{\beta }=\left|C_{2}*X_{\beta }-X\right|$$

(13) $$D_{\delta }=\left|C_{3}*X_{\delta }-X\right|$$

(14) $$X_{1}=X_{\alpha }-A_{1}*D_{\alpha }$$

(15) $$X_{2}=X_{\beta }-A_{2}*D_{\beta }$$

(16) $$X_{3}=X_{\delta }-A_{3}*D_{\delta }$$

(17) $$X\left(t+1\right)=\frac{X_{1}+X_{2}+X_{3}}{3}$$

where $C_{1},C_{2}$, and $C_{3}$ are random vectors; *X* is the ith wolf in the current position vector; $A_{1},A_{2}$ and $A_{3}$ are adaptive vectors; $X_{\alpha }$ represents the current location of the α wolf; $X_{\beta }$ represents the current location of the β wolf; and $X_{\delta }$ is the current location of the δ wolf. Equations (6)–(8) describe the distances between an individual grey wolf and α,β and δ wolves. Equation (12) defines the final position of the grey wolf individual.

When the prey stops moving, grey wolves complete the hunting process by attacking. To simulate encircling of the prey, the value of *a* is gradually decreased, which in turn reduces the fluctuation range of *A*. During the iterations, as the value of *a* decreases linearly from 2 to 0, the corresponding value of *A* varies within the range [−a,a]. As shown in Figure 3, when the value of *A* is within this range, the next position of a grey wolf can be anywhere between its current position and the prey’s position. When |A|<1, the wolves launch an attack on the prey, indicating convergence to a local optimum.

### 4.2. Hybrid Optimization Strategy Combining a Kalman Filter

In the field of optimization algorithms, the alpha (α) wolf in the GWO algorithm is crucial. This study introduces an innovative approach by integrating a Kalman filter into the GWO framework to refine and correct the position of the alpha wolf, enhancing the algorithm’s robustness in complex environments.

The Kalman filter is a recursive algorithm used in real-time control and signal processing to estimate system states and minimize noise. It provides adaptive adjustment, enabling dynamic refinement of the search strategy based on real-time feedback. Combining the global search capabilities of GWO with the adaptive adjustments of the Kalman filter improves the search precision and convergence speed. This hybrid method enhances the optimization efficiency, robustness, and adaptability across various environments.

The process begins with initializing the wolf pack by randomly generating an initial population, each representing a path sequence. Parameters for the Kalman filter, including the state covariance matrix (P), process noise covariance (Q), and observation noise covariance (R), are then initialized. The integration allows for real-time adjustments during the search process, leveraging the strengths of both GWO and the Kalman filter for superior optimization performance. This approach represents a significant advancement, providing a robust, adaptive, and efficient method for solving complex optimization problems in complex systems.

The specific execution steps of the KGWO algorithm are as follows:

1. Generating new candidate path points $\left(z_{t}\right)$ using the GWO algorithm:

1.1 Initialize the number of grey wolves (N) and the position of each grey wolf. Assuming there are *N* path points, each represented by coordinates (x,y);

1.2 Calculate fitness:

Compute the fitness value for each path point using the fitness function:

Fitness function f(x):

(18) f(x)=pL+qS+rO

Path Length (L):

(19) $$L=\sum_{i=1}^{n-1}d\left(x_{i},x_{i+1}\right)$$

Path Smoothness (S):

(20) $$S=\sum_{i=2}^{n-1}\left|\theta_{i}-\theta_{i-1}\right|$$

Obstacle Avoidance (O):

(21) $$O=\sum_{i=1}^{n}\sum_{j=1}^{m}\max\left(0,d_{\mathrm{safe}\;}-d\left(x_{i},o_{j}\right)\right)$$

where p,q, and *r* are weight parameters; $\theta_{i}$ is the turning angle of the i-th point; n is the number of points on the path; $d\left(x_{i},x_{i+1}\right)$ denotes the Euclidean distance between adjacent points on the path; $d_{\mathrm{safe}\;}$ is the safety distance; $d\left(x_{i},o_{j}\right)$ is the distance from path point $x_{i}$ to obstacle $o_{j}$; and m is the number of obstacles.

1.3 Sort the path points based on fitness values and determine the three best positions for α,β, and δ.

1.4 Update the positions of other path points based on the fitness function.

1.5 Continue fitness calculation, sorting, and position updating until the termination condition has been met.

1.6 The final set of path points forms the new candidate path points $z_{t}$.

2. Predict the next moment’s path position using the Kalman filter:

Predict State:

(22) $$x_{t|t-1}=x_{t-1|t-1}$$

Predict Covariance Matrix:

(23) $$P_{t|t-1}=P_{t-1|t-1}+Q$$

Update the predicted state by incorporating observation values (z_t) to obtain the new path position $x_{t|t}$: Calculate Kalman Gain:

(24) $$K_{t}=P_{t|t-1}{\left(P_{t|t-1}+R\right)}^{-1}$$

Update the predicted state:

(25) $$x_{t|t}=x_{t|t-1}+K_{t}\left(z_{t}-x_{t|t-1}\right)$$

Update the covariance matrix:

(26) $$P_{t|t}=\left(I-K_{t}\right)P_{t|t-1}$$

Compute the fitness function $f\left(x_{t|t}\right)$ and determine if the optimal position α needs to be updated:

Update condition for position α:

(27) $$f\left(x_{t|t}\right)<f\left(\alpha \right)$$

(28) $$\alpha =x_{t|t}$$

### 4.3. Partially Matched Crossover (PMX)

Although the Kalman filter correction mechanism can effectively adjust the positions of path points to optimize the path, its updating process is relatively fixed. In environments with high complexity or inconsistency, it may not be able to quickly adapt to changes, thereby affecting the effectiveness of path planning. By introducing moderate random crossover and mutation, the PMX operation can increase the adaptability and evolutionary potential of the population, enhancing the algorithm’s ability to handle complex and nonlinear problems. Additionally, considering the adaptive adjustment of the correlation coefficient α for the distance of individuals to the optimal point, granting individuals different search speeds, will effectively improve the accuracy and convergence rate of the algorithm.

#### 4.3.1. Selecting Crossover Points

Randomly choose two crossover points, *i* and j(i<j), defining the crossover interval. Let the two parent individuals be P1 and P2, representing the sequence of path points:

(29) $$P1=\left[P_{1,1},P_{1,2},P_{1,3},\cdots P_{1,n}\right]$$

(30) $$P2=[P_{2,1},P_{2,2},P_{2,3},\cdots P_{2,n}]$$

(1) Segment exchange:

Within the crossover interval, the segment from Parent 1 is copied into Offspring 2, and the segment from Parent 2 is copied into Offspring 1. The offspring are denoted as C1 and C2:

(31) $$C1=\left[c_{1,1},c_{1,2},c_{1,3},\cdots c_{1,n}\right]$$

(32) $$C2=\left[c_{2,1},c_{2,2},c_{2,3},\cdots c_{2,n}\right]$$

(2) Resulting Offspring:

(33) C1[i:j]=P2[i:j]

(34) C2[i:j]=P1[i:j]

**Figure 4:**
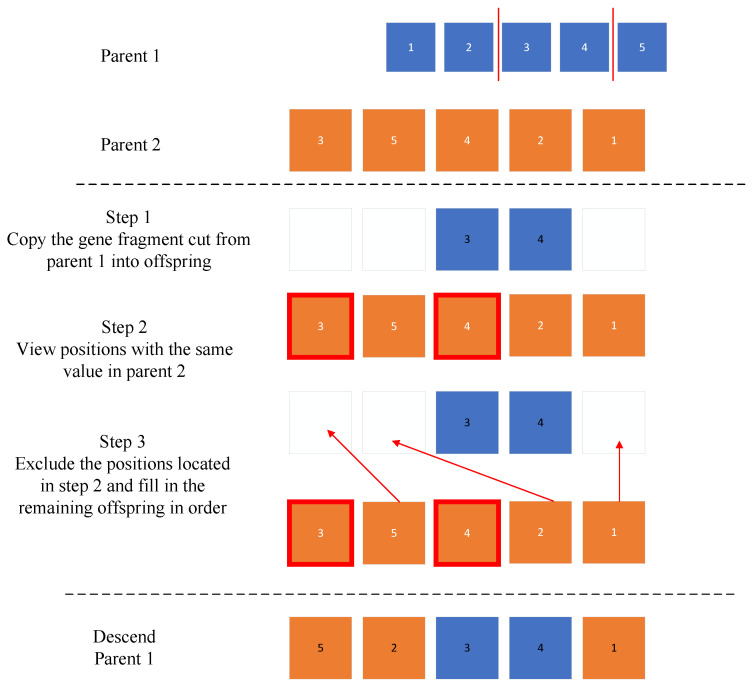
Partially matched crossover (PMX) principle diagram.

To facilitate a clearer understanding of this step, we replace the abstract notation $\left[P_{1,1},P_{1,2},P_{1,3},\cdots P_{1,n}\right]$ and $\left[P_{2,1},P_{2,2},P_{2,3},\cdots P_{2,n}\right]$ with specific numbers, as shown in Figure 4.

#### 4.3.2. Repairing Mapping

After crossover points have been selected, the map is repaired. For each city in the offspring, check if it lies outside the crossover interval. If a duplicate city is found in the offspring, replace it with an unseen city using a predefined mapping to ensure the uniqueness in the path for each city.

(1) Repairing offspring 1:

For offspring 1C1, traverse its segments outside the crossover interval [i:j]. If a city $c_{1,k}$ exists at these positions that duplicates a city within the crossover interval, replace it with the corresponding city from mapping P1.

(2) Repairing offspring 2:

For offspring 2C2, traverse its segments outside the crossover interval [i:j]. If a city $c_{2,k}$ exists at these positions that duplicates a city within the crossover interval, replace it with the corresponding city from mapping P2.

### 4.4. Roulette Wheel Selection

After the partially matched crossover (PMX) operation, there are still certain shortcomings in the selection of parent individuals, which may lead to the underutilization of superior individuals. To address this issue, this paper introduces a roulette wheel selection mechanism. Roulette wheel selection is a mechanism that selects individuals proportionally, with the probability being directly proportional to the individual’s fitness value. When selecting parents, this ensures that individuals with higher fitness values have a greater probability of being selected.

Firstly, calculate the selection probability $P_{i}$ for each individual:

(35) $$P_{i}=\frac{f(i)}{\sum_{j=1}^{N}f\left(j\right)}$$

where f(i) represents the fitness value of the *i*-th individual. Secondly, generate the cumulative probability cum _*p*_ rob _*i*_:

(36) $$\;{\mathrm{cum}\;}_{p}{\mathrm{rob}}_{i}=\sum_{j=1}^{i}p_{j}$$

Then, randomly generate a number *r* in the range ([0,1]), and select the first individual ${\mathrm{cum}\;}_{p}rob_{i}$ that satisfies ${\mathrm{cum}}_{p}rob_{i}\geq r$.

By using this selection mechanism, individuals with higher fitness values have a higher probability of being selected, accelerating the transmission of superior genes. This selection strategy not only effectively enhances the algorithm performance in avoiding local optima but also improves the algorithm’s convergence speed.

**Figure 5:**
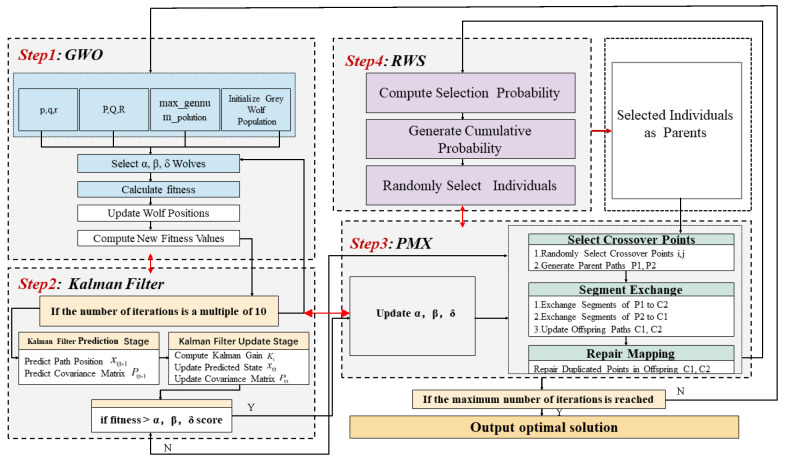
Overall framework of KGWO algorithm.

The overall framework of the proposed method is illustrated in Figure 5, and the detailed implementation process is summarized in Algorithm 1.
**Algorithm 1:** KGWO Algorithm1:**Input:** PopulationSize: Number of wolves, MaxIterations: Maximum number of iterations, KalmanParameters: Initial Kalman filter parameters, ConvergenceThreshold: Threshold for convergence2:**Output:** BestPath: Best path found, BestSequence: Best sequence found3:Initialize the wolf population with *PopulationSize*4:Set initial Kalman filter parameters using *KalmanParameters*5:**while** iterations < MaxIterations and fitness > ConvergenceThreshold **do**6:   Generate candidate path points using GWO algorithm7:   Initialize wolf positions8:   Compute fitness values for each wolf9:   Select alpha, beta, and delta wolves based on fitness10:  Update wolf positions based on alpha, beta, and delta11:  Compute new fitness values for updated positions12:  **Kalman filter prediction stage:**13:  $x_{\mathrm{pred}}\leftarrow$ PredictPathPosition(currentState, KalmanParameters)14:  $P_{\mathrm{pred}}\leftarrow$ PredictCovarianceMatrix(currentCovariance, KalmanParameters)15:  **Kalman filter update stage:**16:  K← ComputeKalmanGain($P_{\mathrm{pred}}$, measurementNoise)17:  $x_{t|t}\leftarrow$ UpdatePredictedState($x_{\mathrm{pred}}$, *K*, measurement)18:  $P_{\mathrm{upd}}\leftarrow$ UpdateCovarianceMatrix($P_{\mathrm{pred}}$, *K*, measurementNoise)19:  Compute fitness function for updated state20:  $f(x_{t|t})\leftarrow$ ComputeFitness($x_{t|t}$)21:  **if** $f\left(x_{t|t}\right)<f\left(\alpha \right)$ **then**22:      Update α to $x_{t|t}$23:  **else**24:      Continue25:  **end if**26:  Select crossover points i,j randomly27:  Generate parent paths $P_{1}$, $P_{2}$28:  Perform segment exchange between $P_{1}$ and $P_{2}$ to generate $C_{1}$, $C_{2}$29:  Repair mappings of $C_{1}$, $C_{2}$ to ensure valid paths30:  Apply roulette wheel selection mechanism31:  Compute selection probabilities $P_{i}$ for each wolf32:  Compute cumulative probabilities com_prob_*i*_33:  Perform roulette wheel selection to choose individuals for next generation34:  Generate new population using selected individuals35:  **if** MaxIterationsReached() or ConvergenceConditionMet() **then**36:      **OUTPUT:** BestPath and BestSequence37:      **EXIT**38:  **else**39:      Continue40:  **end if**41:**end while**42:**Return:** BestPath, BestSequence

### 4.5. Algorithm Testing with Benchmark Functions

To validate the effectiveness of the proposed Kalman filter–grey wolf optimization (KGWO) algorithm, six well-known optimization algorithms were utilized for comparison: particle swarm optimization (PSO), grey wolf optimization (GWO), Genetic Algorithm (GA), whale optimization algorithm (WOA), sine cosine algorithm (SCA), and artificial bee colony (ABC). The experimental parameters were uniformly set across all algorithms to ensure a fair comparison. The population size was set to 30, and the number of iterations was set to 500. To minimize the experimental randomness, all algorithms were independently run 20 times, and the average results were taken. The convergence accuracy is represented by the average value of the 20 runs, and the stability of the algorithms is indicated by the standard deviation of these results.

The benchmark functions used are described as follows:

F5 (Sphere Function): A simple unimodal function used to test basic optimization capabilities.

F6 (Rosenbrock Function): A unimodal function with a narrow, curved valley, testing the algorithm’s local search capability.

F8 (Shifted Ackley’s Function): A classic multimodal optimization problem designed to evaluate an algorithm’s ability to handle complex global structures.

F10 (Shifted Rotated Rastrigin’s Function): A variant of the Rastrigin function, frequently used to assess the robustness and performance of optimization algorithms.

F12 (Rotated Rastrigin Function): A complex multimodal function with periodic peaks, challenging an algorithm’s robustness in high-dimensional spaces.

F13 (Expanded Scaffer’s F6 Function): A highly complex multimodal function that tests an algorithm’s ability to handle nonlinear and rugged search spaces.

A detailed description of the abovementioned benchmark functions is listed in Table 1.

**Figure 6:**
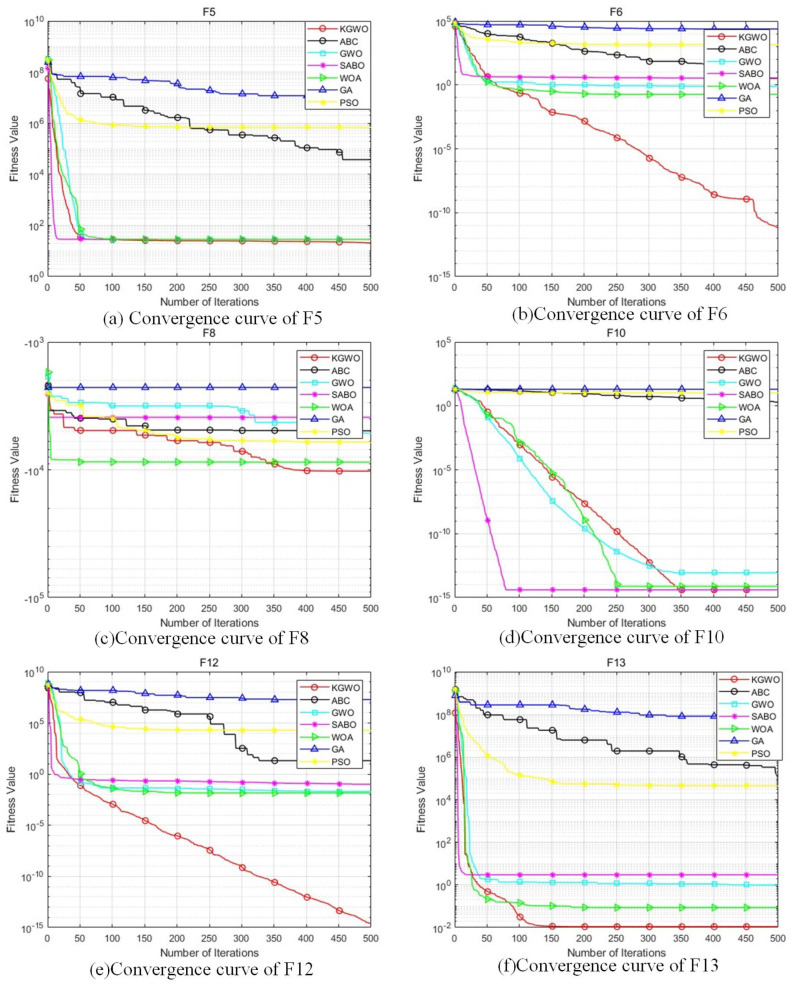
Comparison of fitness values and convergence curves of test functions (from (**a**–**f**): F5, F6, F8, F10, F12, and F13).

The testing results are shown in Table 2 and Figure 6, which indicate that KGWO consistently outperformed the other six algorithms across all benchmark functions. Specifically:

1. Convergence Accuracy: KGWO achieved the lowest average objective function values on all test functions, indicating its superior ability to find optimal or near-optimal solutions. This suggests that the integration of Kalman filtering with GWO enhanced the precision of path planning.
2. Stability: The standard deviation of KGWO’s results was consistently lower than those of the other algorithms, demonstrating a higher reliability and robustness. This highlights KGWO’s effectiveness in maintaining consistent performance across multiple runs.
3. Convergence Speed: The convergence curves in Figure 6 show that KGWO reached optimal solutions faster than the other algorithms. This can be attributed to the algorithm’s hybrid approach, which combines the exploration capabilities of GWO with the refinement abilities of Kalman filtering and PMX operations.
4. Handling Complex Functions: For multimodal functions like Shifted Ackley’s and Expanded Scaffer’s F6, KGWO exhibited significant improvements in finding the global optimum, while avoiding local optima. This underscores the algorithm’s capability to navigate complex and rugged search spaces effectively.

In summary, the KGWO algorithm demonstrated superior performance in terms of accuracy, stability, and convergence speed compared to the PSO, GWO, GA, WOA, SCA, and ABC algorithms. The enhancements introduced by incorporating a Kalman filter, elite strategy, and PMX mutation operations enabled KGWO to efficiently tackle complex path planning problems, making it a robust and reliable solution for AGV navigation in intricate environments.

## 5. Path Planning Simulation

To validate the adaptability and superiority of the proposed KGWO algorithm, two distinct scenarios within the context of an automated warehouse environment were used: Case 1: densely packed goods scenario; Case 2: complex indoor layout scenario.

The performance of the KGWO algorithm was compared against three other algorithms: grey wolf optimization (GWO), improved grey wolf optimization (IGWO), and adaptive grey wolf optimization (AGWO). These four algorithms were chosen due to their distinctive features: GWO mimics the social hunting behavior of grey wolves, balancing local and global search capabilities; IGWO enhances GWO with dynamic adjustments and additional strategies for improved performance; AGWO introduces adaptive mechanisms for dynamic parameter adjustment during the search process, enhancing the robustness and adaptability; and KGWO integrates K-means clustering with GWO to improve the initial solution quality and balance between local and global search. All four algorithms were tested, starting from the same initial point and reaching the same destination, allowing a comprehensive comparison of their path planning results.

The simulation studies were conducted in MATLAB (R2022a) under Windows 11 on a laptop with a 2.50 GHz AMD Ryzen 5 4600U CPU and 32.0 GB RAM.

The comparison and analysis focused on three main aspects: path length, the number of convergence iterations, and the execution time of the algorithm. In this study, “path length” refers to the total distance the robot travels. A shorter path is preferable as it indicates higher efficiency, lower energy consumption, and reduced operational costs, which are crucial for optimizing the performance of AGVs in industrial settings.

“Convergence iterations” represent the number of cycles the algorithm needed to reach an optimal or near-optimal solution. Fewer iterations are considered better because they demonstrate the algorithm’s ability to find a solution quickly, reflecting its efficiency in handling complex environments. A high convergence speed is essential for real-time applications, where quick decision-making is necessary.

“Execution time” measures the total computational time required by the algorithm to generate a path. For real-time applications, such as in dynamic or unpredictable environments, shorter execution times are critical, as they enable the AGV to respond promptly to changes and ensure smooth operation.

In the context of this paper, a good algorithm would minimize both the path length and execution time, while requiring fewer convergence iterations. These criteria collectively evaluate the balance between the algorithm’s efficiency (shorter paths and quick computation) and its effectiveness (fewer iterations indicating faster convergence and robustness in diverse scenarios). The choice of these metrics stemmed from their direct relevance to evaluating the algorithm’s applicability and performance in practical, real-world scenarios.

The initial parameters of the algorithm were chosen based on a combination of empirical studies, a literature review, and preliminary testing [35,36]. They provide guidelines for parameter settings in optimization algorithms. Specifically, the maximum number of iterations ($N_{\max}$) was set to 500, to balance the convergence speed and solution quality, allowing the algorithm sufficient time to explore the search space, while preventing excessive computational cost. The population size (*m*) was chosen as 100, a standard value that has proven effective in various optimization problems, providing a good trade-off between exploration and exploitation. The dimensionality of the search space (dim) was set to 2, corresponding to the 2D nature of the path planning problem in this study. Parameters *p*, *q*, and *r* represent algorithm-specific probabilities or factors, which were based on typical values used in similar optimization algorithms, to ensure the algorithm’s robustness across different scenarios. These parameters were fine-tuned during the initial testing, to ensure optimal performance in the specific industrial environment of the AGV.

Detailed initial algorithm parameters are listed in Table 3.

### 5.1. Simulation Case 1: Densely Packed Goods Scenario

**Figure 7:**
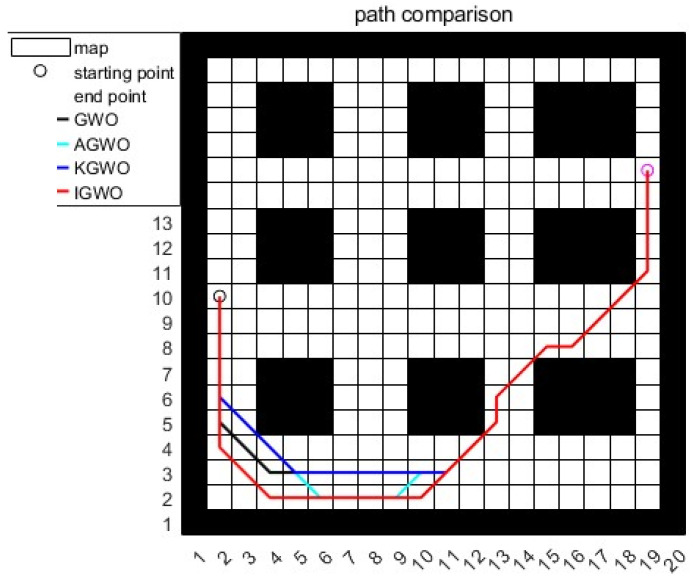
Path planning results for simulation case 1.

**Figure 8:**
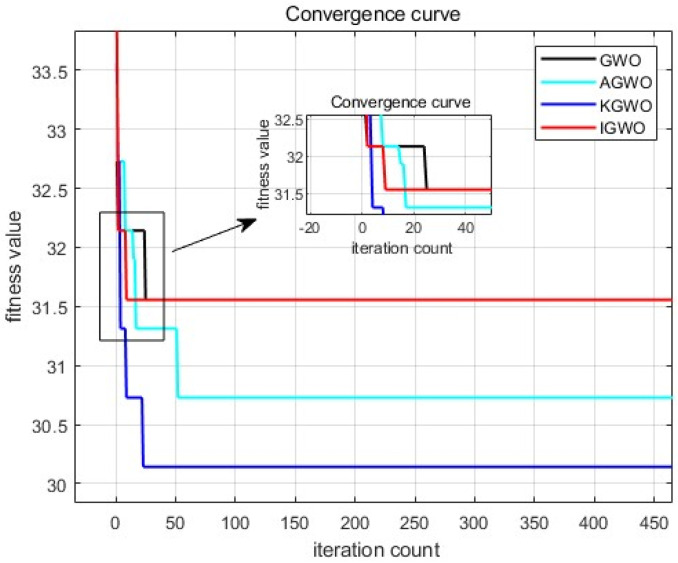
Iteration graph of route length for simulation case 1.

A densely packed goods scenario was utilized for path planning in case 1, with the simulation results presented in Figure 7 and Figure 8 and Table 4. The simulation results indicated that the traditional GWO algorithm struggled with global path optimization, often getting stuck in local optima and yielding unstable path values across multiple experiments. This was primarily due to premature convergence towards the positions of α,β, and δ wolves, which limited the exploration of the search space.

The IGWO algorithm improved the early-stage convergence speed and reduced the likelihood of falling into local optima. However, its emphasis on rapid convergence through dynamic contraction factors led to suboptimal shortest path results in complex environments. The AGWO algorithm significantly reduced the path length using adaptive weighting mechanisms but failed to enhance the local search capabilities when encountering obstacles, resulting in prolonged stagnation in local optima and a slower convergence.

In contrast, the KGWO algorithm, integrating Kalman filter and probability model crossover (PMX) operations, effectively avoided local optima from the 24th generation onward. This reduces the local stagnation and improved the overall search performance, as depicted in Figure 8. KGWO reduced the path length by 4.29 m (13.6%) compared to traditional GWO, 1.29 m (4.1%) compared to IGWO, and 0.53 m (1.7%) compared to AGWO. In terms of simulation time, KGWO was 0.12 s faster than traditional GWO, 0.09 s faster than IGWO, and 0.69 s faster than AGWO. These improvements highlight the KGWO algorithm’s enhanced stability and efficiency in dense, obstacle-rich environments.

Furthermore, the KGWO algorithm significantly enhanced the path accuracy by ensuring the AGV followed a trajectory that closely aligned with the optimal path, minimizing deviations, even in the presence of densely packed obstacles. This improvement is crucial for applications requiring high precision in navigation. The stability of KGWO was demonstrated through a consistent convergence behavior across multiple trials, reducing the variance in path length and improving the reliability under varying conditions. Moreover, the diversity of solutions generated by KGWO across the different runs underscored its robust exploration capabilities, preventing premature convergence and ensuring a comprehensive search of the solution space. These enhancements collectively contribute to more reliable and precise path planning in complex environments.

### 5.2. Simulation Case 2: Complex Indoor Layout Scenario

**Figure 9:**
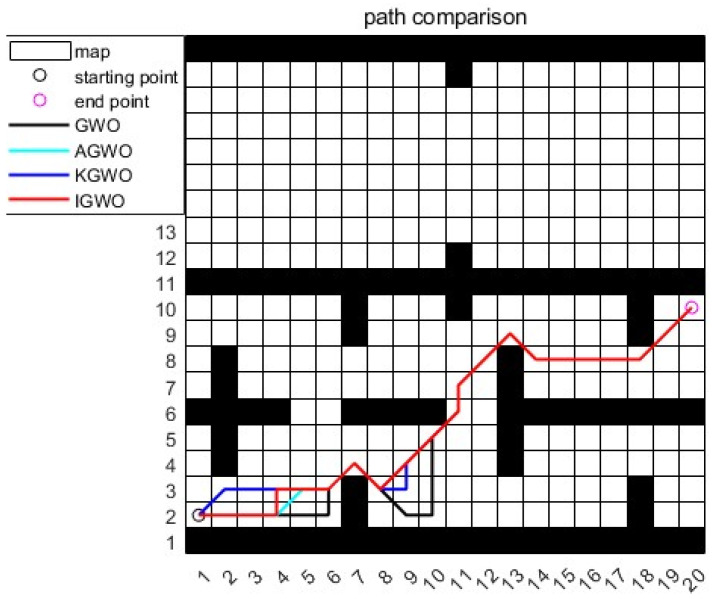
Path planning results for simulation case 2.

**Figure 10:**
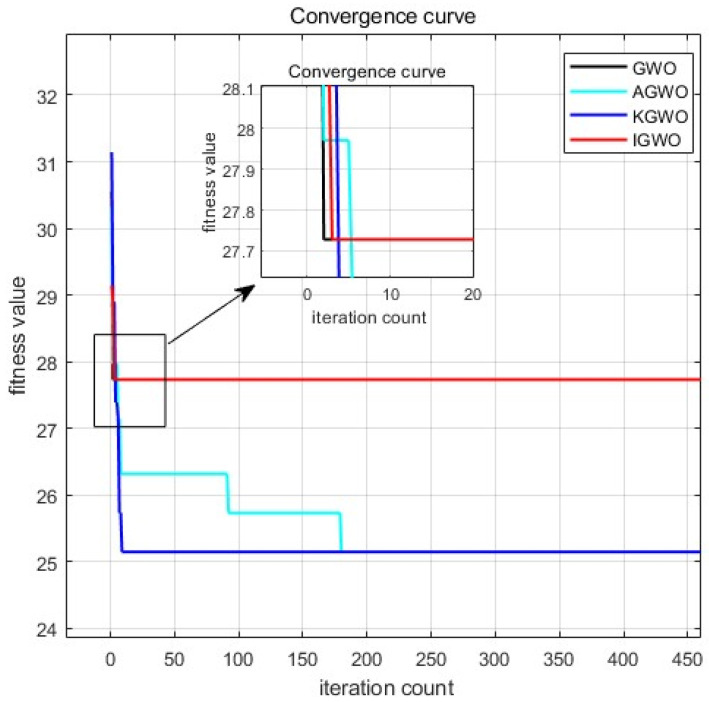
Iteration graph of route length for simulation case 2.

The second scenario, also within the Automated Warehouse Environment, featured a complex indoor layout, as shown in Figure 9. The simulation results are presented in Figure 9 and Figure 10 and Table 5. This setup included partitions and obstacles, simulating environments such as factories or office buildings where AGVs must navigate through restricted spaces.

In case 2, the traditional GWO algorithm exhibited a fast convergence but produced suboptimal path results, due to premature convergence towards α,β, and δ wolves. The IGWO algorithm showed a rapid earlystage convergence but struggled to improve the path distance in obstacle-rich environments, only achieving the global optimal path convergence in the 364th generation. The AGWO algorithm accelerated the initial convergence and reduced the path length but experienced extended local convergence periods, failing to overcome local optima effectively.

The KGWO algorithm excelled by escaping local extrema and identifying the global optimal path as early as the 6th generation, reducing local stagnation time, improving convergence speed, and minimizing path distance. This superior performance was facilitated by Kalman filtering for enhanced path prediction, and PMX operations for robust exploration of the solution space.

According to Table 5, KGWO reduced the path length by 2.68 m (9.6%) compared to traditional GWO, by 2.68 m (9.6%) compared to IGWO, and by 3.68 m (13.8%) compared to AGWO. In terms of simulation time, KGWO decreased this by 0.01 s compared to GWO, 0.02 s compared to IGWO, and 2.71 s compared to AGWO. The iteration count for convergence with KGWO notably decreased to 19 iterations, compared to 20 for GWO and 169 for AGWO.

These findings underscored the KGWO algorithm’s ability to integrate advanced search strategies, significantly enhancing the overall convergence performance, search efficiency, and obstacle avoidance capabilities in complex automated warehouse environments.

### 5.3. Simulation Case 3: Adaptability Analysis on Random Maps

**Figure 11:**
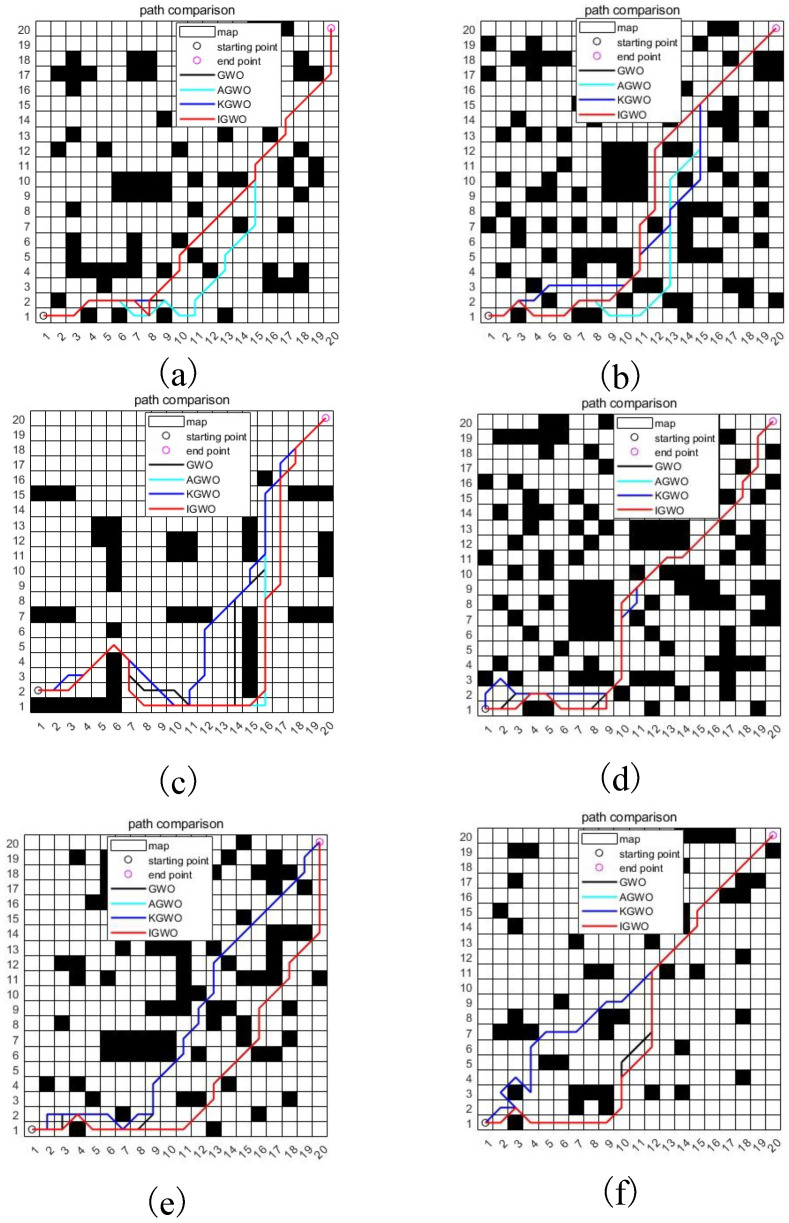
Path planning results for simulation case 3. (**a**) Random map 1. (**b**) Random map 2. (**c**) Random map 3. (**d**) Random map 4. (**e**) Random map 5. (**f**) Random map 6.

To further validate the adaptability and superiority of KGWO in path planning, six maps were randomly selected for testing in this case. The simulation results are presented in Figure 11 and Table 6. The data analysis results show that KGWO converged 21.78% faster than traditional GWO, 25.79% faster than IGWO, and 22.69% faster than AGWO. In terms of path length, the KGWO’s paths were 6.99% shorter than GWO, 9.55% shorter than IGWO, and 3.48% shorter than AGWO. These results demonstrate that KGWO not only improved the convergence speed but also optimized the path length in different maps, showcasing its superiority and adaptability in complex and random environments.

Furthermore, the KGWO algorithm significantly enhanced the path accuracy by ensuring the AGV followed a trajectory that closely aligned with the optimal path, minimizing deviations, even in the presence of densely packed obstacles. This improvement is crucial for applications requiring high precision in navigation. The stability of KGWO was demonstrated through a consistent convergence behavior across multiple trials, reducing the variance in path length and improving the reliability under varying conditions. Moreover, the diversity of solutions generated by KGWO across the different runs underscored its robust exploration capabilities, preventing premature convergence and ensuring a comprehensive search of the solution space. These enhancements can collectively contribute to more reliable and precise path planning in complex environments.

## 6. Experiment

**Figure 12:**
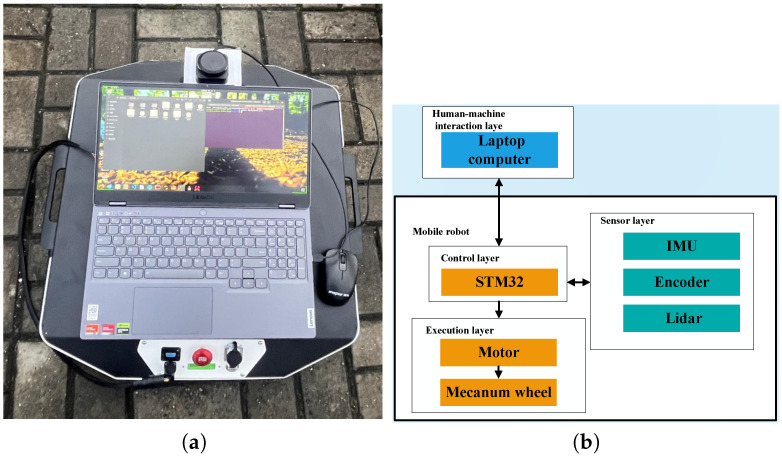
The robot and hardware system used in the experiment. (**a**) The “TRACER” robot connected to the radar and the computer used as the host. (**b**) The hardware system.

To validate the superiority and effectiveness of the proposed algorithm in real-world scenarios, experiments were conducted on a real AGV (automated guided vehicle) using the ROS platform. The experiments utilized the TRACER mobile robot developed under the guidance of Songlin LiDAR, as shown in Figure 12a. The robot was modified to communicate via CAN and equipped with an RPLIDAR S3 from Slamtec, featuring a 40-m measurement radius, 32 k sampling frequency, and 20 Hz scanning frequency. These modifications and advanced sensors were crucial for accurately testing the performance of the proposed algorithm under real operating conditions, ensuring the relevance and applicability of the results obtained from the simulations.

As shown in Figure 12b, the hardware system architecture comprised control, sensing, execution, and human–machine interaction layers. These layers work together seamlessly during navigation tasks. Commands from the human–machine interaction layer are sent to the control layer of the mobile robot. The control layer processes these commands, along with environmental perception data from the sensing layer and self-state information, controlling the execution layer’s motion in real-time via serial communication to complete tasks such as map creation and path planning.

The software system was developed based on the distributed framework of ROS, aiming to achieve motion control of the mobile robot through navigation commands. The experimental site was set up in an outdoor environment, using plastic boards to build fences and obstacles. Considering the hardware capabilities and experimental conditions, the SLAM_HECTOR module framework was chosen to construct the navigation grid. This framework requires relatively low computational resources, while providing high accuracy.

In real-world scenarios, the environment is more complex and richer in information compared to simulation environments. To ensure the accuracy of the results, relevant parameters were set to avoid configuration issues that could lead to navigation failures. The key parameter configurations are listed in Table 7.

### 6.1. The Impact of Algorithm Iteration Counts on Experiments in Real-World Environments

To verify whether changes in the number of iterations affected the longest path and experiment time of the algorithms, experiments were conducted using the four algorithms with the same population size in the environments depicted in Figure 1b,d.

The experiments were conducted at the aquaculture base of Huazhong Agricultural University, where the testing environment was designed to simulate a factory-based warehouse setting. Partitions and barriers were strategically placed to mimic real-world obstacles, and the entire layout was mapped using the simultaneous localization and mapping (SLAM) method. To capture a comprehensive view of the environment, drones were used for aerial photography, which allowed for a detailed overview of the setup. Key obstacles were marked with red lines in the generated map, to enhance clarity and facilitate the study. This map was then utilized for path planning experiments by integrating it into the navigation system.

**Figure 13:**
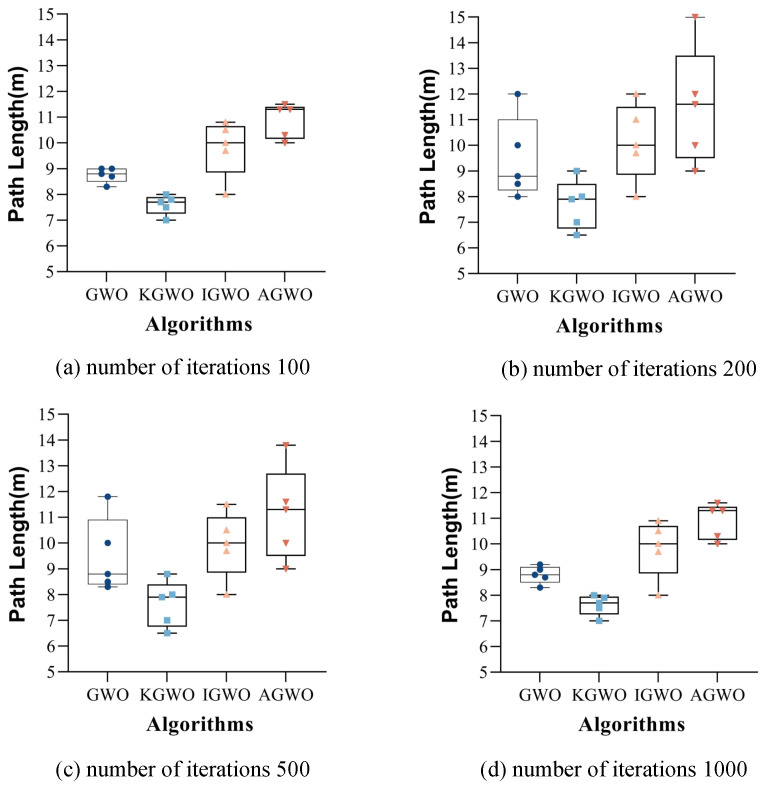
Path length comparison in densely packed goods scenario across iterations.

**Figure 14:**
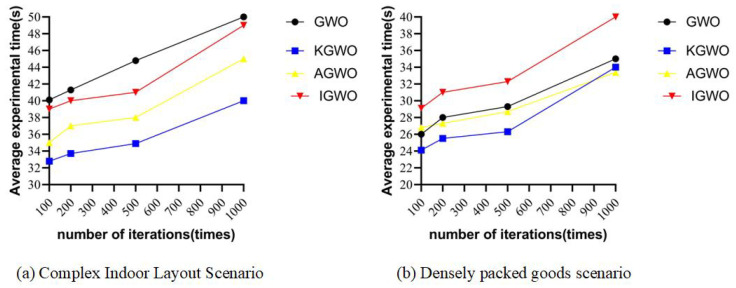
Iteration time comparison across environments and iterations.

The number of iterations was set to 100, 200, 500, and 1000, respectively, with five sets of experiments conducted for each iteration count. The results are presented in Figure 13 and Figure 14.

From Figure 13 and Figure 14, it can be observed that over 500 iterations, the differences in extremum values among the four algorithms were small, and their average values were relatively low, resulting in favorable path outcomes. Compared to experiments with 1000 iterations, the experiment duration was shorter with 500 iterations. Based on these observations, 500 iterations were selected for the real-world experiments. This choice allowed the four algorithms to achieve relatively optimal results, while also reducing the overall experiment time.

### 6.2. Experiment Case 1: Complex Indoor Layout Scenario

**Figure 15:**
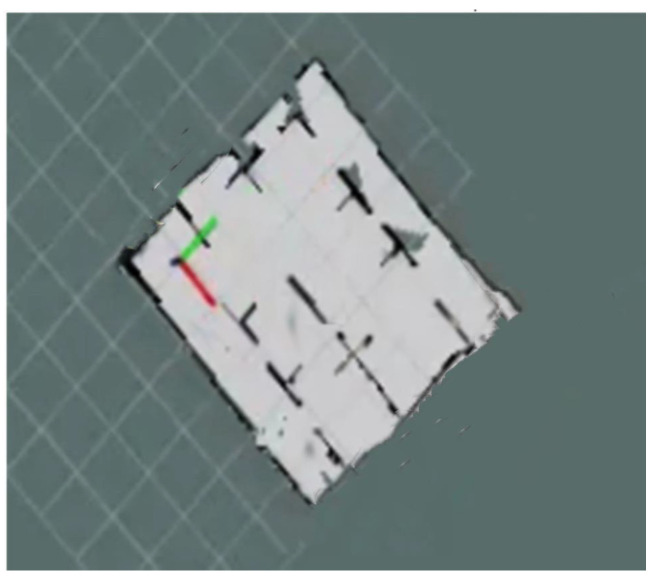
Complex indoor layout scenario map using ROS SLAM.

**Figure 16:**
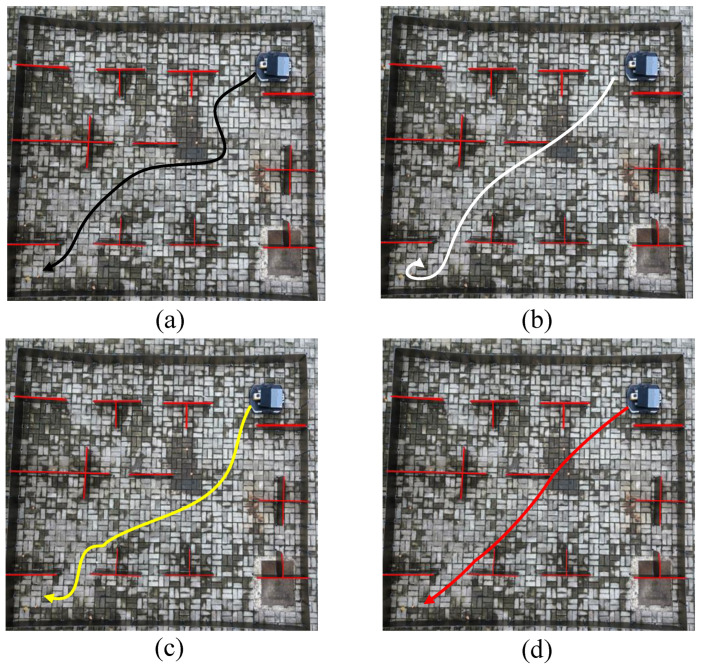
The actual path planning results of four algorithms. (From (**a**–**d**): GWO, IGWO, AGWO, and KGWO).

**Figure 17:**
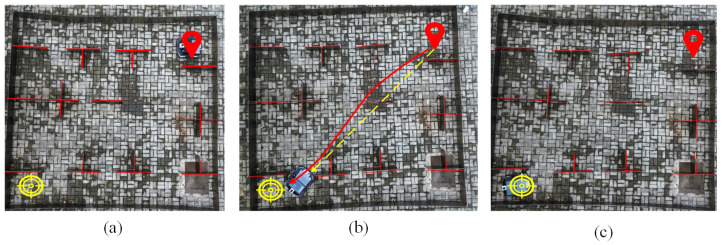
The moving process of the robot. (From (**a**–**c**): the start, through the planned path, and the final destination).

In experiment case 1, a complex indoor layout scenario was constructed, whose map was created using the simultaneous localization and mapping method (SLAM), as shown in Figure 15. The saved static map was transmitted to the path planning module in node form to conduct path planning experiments on the mobile robot. The experimental results of case 1 are shown in Figure 16 and Figure 17 and Table 8.

Figure 16a presents the path planned by the traditional Grey Wolf Optimizer (GWO) algorithm. This path exhibits numerous sharp turns, excessive angles, and longer route lengths. These characteristics arise from the GWO algorithm’s limited local search capabilities, which predispose it to local optima, particularly in environments densely populated with obstacles. Here, the alpha (α), beta (β), and delta (δ) wolves often converge within restricted local regions, thus restricting the exploration scope.

In contrast, Figure 16b demonstrates the enhanced path planning achieved by the Improved Grey Wolf Optimizer (IGWO). IGWO reduces the number of turns and ensures smoother paths through improved global search capabilities enabled by dynamic contraction factors and disturbance strategies. Despite these advancements, IGWO may still exhibit larger turning angles when maneuvering close to obstacles due to the absence of specialized optimizations tailored for obstacle avoidance.

Similarly, Figure 16c depicts the path planned by the adaptive grey wolf optimizer (AGWO), which introduced adaptive weighting mechanisms to adjust search strategies based on environmental complexity. AGWO mitigates some challenges associated with traditional GWO but may require significant turning angles near obstacles, reflecting its general adaptability, without specialized obstacle-handling optimizations.

Finally, Figure 16d illustrates the paths generated by the KGWO algorithm studied in this research. KGWO integrates Kalman filter and probability model crossover (PMX) operations, significantly enhancing prediction accuracy and path planning robustness in complex dynamic environments. As shown in Table 8, this approach reduced the average path length by 1.56 m, decreased the time spent by 5.6 s, and minimized the number of turns, resulting in smoother paths. These improvements are attributed to KGWO’s superior adaptability and prediction capabilities in dynamic environments, thereby overcoming limitations observed in the previous GWO, IGWO, and AGWO algorithms.

In summary, KGWO represents a substantial advancement by effectively combining a Kalman filter for precise state estimation and PMX for enhanced path diversity, achieving superior path planning efficiency and accuracy compared to its predecessors.

Figure 17a, Figure 17b, and Figure 17c, respectively, show the process of the robot from the start, through the planned path, to the final destination using KGWO as the global path planning algorithm. In Figure 17b, it is evident that throughout the robot’s movement, the angles did not change significantly, and the path was very smooth. This demonstrates the significant advantages of the improved KGWO algorithm for AGV path planning in an unmanned automated facility environment. The smoothness of the path and minimal angle changes highlight the algorithm’s efficiency in optimizing the path accuracy and stability, key performance metrics that were enhanced by the incorporation of Kalman filter corrections into the grey wolf optimization framework.

### 6.3. Experiment Case 2: Densely Packed Goods Scenario

**Figure 18:**
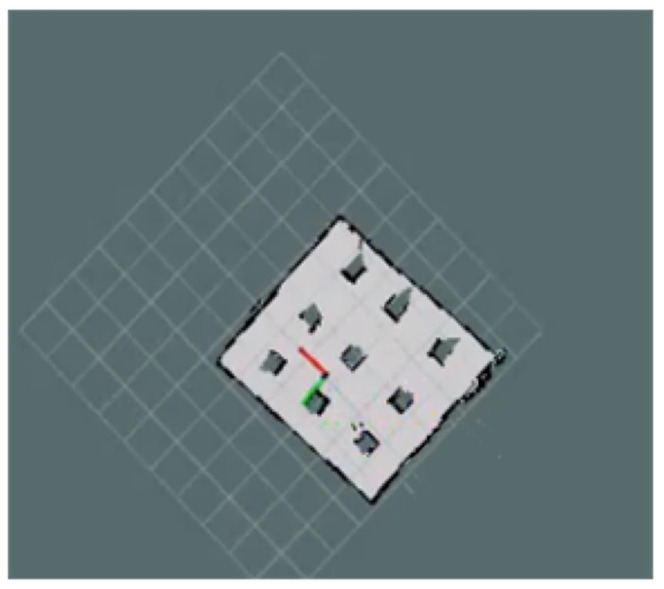
Densely packed goods scenario map using ROS SLAM.

**Figure 19:**
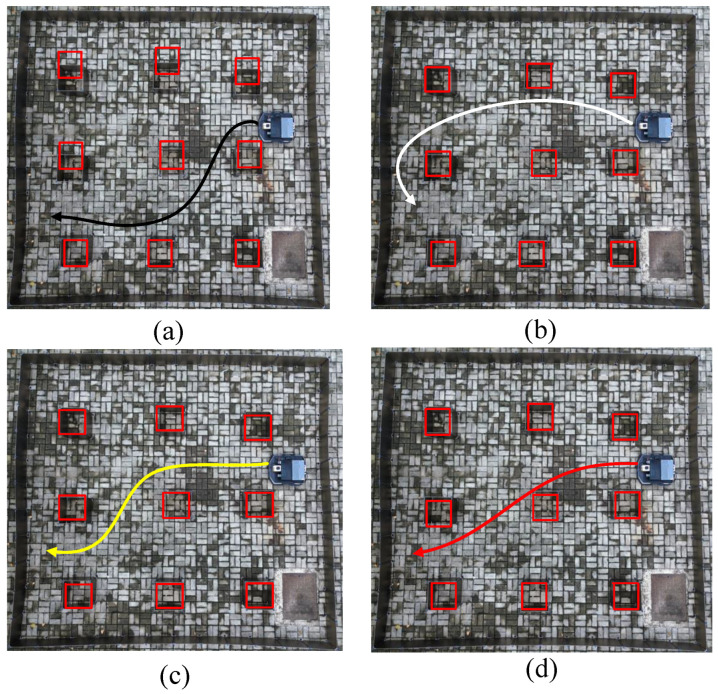
The actual path planning results of the four algorithms. (From (**a**–**d**): GWO, IGWO, AGWO, and KGWO).

**Figure 20:**
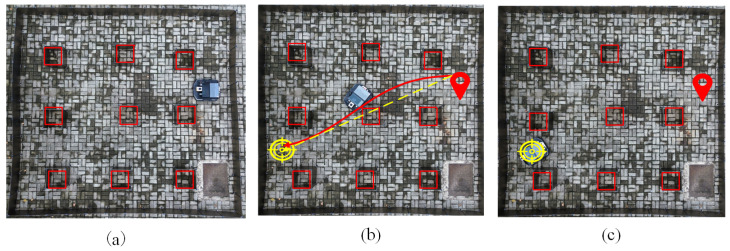
The process of the robot (from (**a**–**c**): the start, through the planned path, and final destination).

In experiment case 2, a densely packed goods scenario was constructed. The environment map is shown in Figure 18, and the experiment results are shown in Figure 19 and Figure 20 and Table 7.

As shown in Figure 19a, the path planned by the traditional GWO algorithm exhibited more turning points, excessive angles, and longer routes. This was due to its limited local search capability in complex environments, leading to susceptibility to local optima.

As shown in Figure 19b,c, both the IGWO and AGWO algorithms showed a reduction in turning points and relatively smoother paths. However, they exhibited larger angles near obstacles, indicating their improved global search capabilities but continued challenges in obstacle handling adaptation. As shown in Table 9, the KGWO algorithm studied in this paper achieved an average path length reduction of 1.23 m and a time reduction of 3.6 s compared to the traditional methods. Moreover, it significantly reduced the number of turns and ensured smoother paths. This improvement is attributed to the algorithm’s Kalman-filter-based prediction mechanism and probabilistic model crossover operation, which enhance both the local and global search capabilities in complex dynamic environments.

Figure 20 illustrates the dynamic process of an automated guided vehicle (AGV), starting from its initial position, navigating through obstacles, and successfully reaching its destination under the guidance of KGWO. It is evident from the figure that under the algorithm’s guidance, the AGV adeptly and efficiently maneuvered around obstacles, showcasing the effectiveness of the KGWO algorithm in optimizing AGV path planning. The AGV’s ability to smoothly navigate around obstacles, as observed in the figure, underscores the algorithm’s enhanced stability and path diversity. These improvements stem from the KGWO’s dynamic adjustment mechanisms and predictive capabilities, which are crucial in addressing challenges related to path accuracy and stability.

## 7. Conclusions

This study presented a hybrid optimization method that combines a Kalman filter with grey wolf optimization (GWO) to address the challenges of automated guided vehicle (AGV) path planning. The proposed approach significantly enhances path accuracy, stability, and diversity by generating initial paths using GWO and periodically refining them with Kalman filter corrections, ensuring precise and stable navigation. The inclusion of roulette wheel selection, an elite strategy, and partially matched crossover (PMX) further boosts the evolutionary process, producing diverse and high-quality paths.

Experimental results from two scenarios within automated warehouse environments (a densely packed storage setup and a complex indoor layout) demonstrated the superior performance of the proposed algorithm. The hybrid method showed notable improvements in speed and path length compared to conventional methods. It not only enhanced the optimization metrics but also ensured more predictable and collision-free navigation paths, particularly in environments with static obstacles. This improves operational efficiency and safety, highlighting the method’s potential in real-world applications such as automated distribution centers and unmanned parking facilities.

The integration of the Kalman filter with GWO offers a robust solution for AGV path planning, addressing the limitations of traditional algorithms and providing a practical approach for complex unmanned environments. The findings represent a significant advancement in the field of mobile robot intelligence, promoting the efficiency and reliability of AGVs in modern logistics and industrial automation.

However, certain limitations remain. The proposed method may face challenges in highly dynamic environments where obstacles are frequently moving. Additionally, the computational complexity of the hybrid method, particularly when running on systems with limited CPU power and memory, such as a typical laptop, could lead to longer execution times and difficulties in handling large datasets. Further research is needed to optimize the algorithm’s performance in dynamic scenarios and reduce its computational load for real-time applications.

## Figures and Tables

**Table 1:** Benchmark function description.

Functions	Dim	Range	$f_{\min}$
$F_{5}\left(x\right)=\sum_{i=1}^{n}x_{i}^{2}$	30	[−30,30]	0
$F_{6}\left(x\right)=\sum_{i=1}^{n-1}\left[100{\left(x_{i+1}-x_{i}^{2}\right)}^{2}+{\left(1-x_{i}\right)}^{2}\right]$	30	[−1.28,1.28]	0
$F_{8}\left(x\right)=-20\exp\left(-0.2\sqrt{\frac{1}{n}\sum_{i=1}^{n}x_{i}^{2}}\right)-\exp\left(\frac{1}{n}\sum_{i=1}^{n}\cos\left(2\pi x_{i}\right)\right)+20+e$	30	[−5.12,5.12]	0
$F_{10}\left(x\right)=\sum_{i=1}^{n}\left[{\left(x_{i}-o_{i}\right)}^{2}-10\cos\left(2\pi \left(x_{i}-o_{i}\right)\right)+10\right]$	30	[−600,600]	0
$F_{12}\left(x\right)=\sum_{i=1}^{n}\left[x_{i}^{2}-10\cos\left(2\pi x_{i}\right)+10\right]$	4	[−5,5]	0.0003
$F_{13}=0.5+\frac{\sin^{2}\left(\sqrt{x_{1}^{2}+x_{2}^{2}}\right)-0.5}{{\left[1+0.001\left(x_{1}^{2}+x_{2}^{2}\right)\right]}^{2}}$	2	[−5,5]	−1.031

**Table 2:** Results of algorithm testing.

Function	Performance	KGWO	GWO	ABC	SABO	WOA	GA	PSO
$F_{5}$	mean	21.2123	27.2293	120,500.9477	28.5985	27.6793	22,725,509.5085	309,705.93
	std	1.1686	1.0379	39,145.855	0.406520	0.21665	12,572,125.086	201,800.3946
$F_{6}$	mean	2.0471 × 10^−13^	0.75379	3.5646	2.9153	0.56014	26,853.4907	1665.6832
	std	7.222 × 10^−14^	1.4095	0.24991	0.60604	0.37414	7999.1148	526.8057
$F_{8}$	mean	−9416.1449	−6669.5007	−5044.0135	−3181.825	−10,332.1725	−2090.2389	−6022.3568
	std	1304.3827	349.5092	617.4954	352.5995	1525.7358	739.9496	718.1929
$F_{10}$	mean	5.4179 × 10^−15^	9.2815 × 10^−14^	2.0019	3.9968 × 10^−15^	4.7073 × 10^−15^	19.8359	11.4342
	std	1.9459 × 10^−15^	0.64065	1.0049 × 10^−14^	0	2.9724 × 10^−15^	0.41608	1.7818
$F_{12}$	mean	1.0828 × 10^−14^	0.039896	17.3748	0.25506	0.02838	54,810,209.4934	43.6245
	std	1.1488 × 10^−14^	12.9832	0.0042866	0.043934	0.014687	41,689,636.6304	17.4385
$F_{13}$	mean	0.0042048	0.64112	4274.8115	2.383	0.50851	87,618,066.6031	474,827.8802
	std	0.0094021	0.20879	7921.5278	0.89207	0.29697	53,928,474.9985	731,525.0567

**Table 3:** Initial parameter setup.

Parameters	Value
$N_{\max}$	500
*m*	100
dim	2
*p*	0.5
*q*	0.3
*r*	0.2

**Table 4:** Statistical analysis of experimental data for simulation case 1.

Metrics	Algorithms	Results
Simulation Time (s)	GWO	2.17
	IGWO	1.88
	AGWO	2.74
	KGWO	2.05
Path Length (m)	GWO	31.51
	IGWO	31.51
	AGWO	30.75
	KGWO	30.22
Convergence iterations (times)	GWO	22
	IGWO	9
	AGWO	50
	KGWO	20

**Table 5:** Statistical analysis of experimental data for simulation case 2.

Metrics	Algorithms	Results
Simulation Time (s)	GWO	1.11
	IGWO	1.10
	AGWO	3.83
	KGWO	1.12
Path Length (m)	GWO	27.81
	IGWO	27.81
	AGWO	25.13
	KGWO	25.13
Convergence iterations (times)	GWO	3
	IGWO	4
	AGWO	171
	KGWO	9

**Table 6:** Statistical analysis of experimental data for simulation case 3.

Map	Algorithms	Simulation Time (s)	Path Length (m)
1	GWO	2.37	49.77
	IGWO	3.12	48.51
	AGWO	2.22	48.26
	KGWO	1.83	46.13
2	GWO	1.97	39.98
	IGWO	1.65	38.14
	AGWO	1.69	38.72
	KGWO	1.56	36.74
3	GWO	2.33	42.38
	IGWO	2.03	50.38
	AGWO	2.74	42.97
	KGWO	2.06	40.04
4	GWO	3.22	40.12
	IGWO	2.55	40.38
	AGWO	2.96	35.89
	KGWO	2.51	35.55
5	GWO	2.78	40.55
	IGWO	3.63	40.61
	AGWO	3.01	40.23
	KGWO	2.66	40.07
6	GWO	2.73	42.85
	IGWO	3.25	43.66
	AGWO	3.00	40.41
	KGWO	2.44	38.76

**Table 7:** Robot parameter settings based on ROS.

Parameters	Value
inflation_range	1.0 m
observation_source	/scan
update_frequency	1.0 Hz
max_vel_x	0.3m/s
global_frame	−0.06m/s
publish_frequency	1.0 mz

**Table 8:** Experimental data statistics of experiment case 1.

Algorithm	Path Length (m)	Arrival Time (s)	Number of Turns (Times)
GWO	12.0	40.0	5
IGWO	12.3	41.0	3
AGWO	11.6	38.6	3
KGWO	10.4	34.7	1

**Table 9:** Experimental data statistics of experiment case 2.

Algorithm	Path Length (m)	Arrival Time (s)	Number of Turns (Times)
GWO	8.8	29.3	2
IGWO	9.7	32.3	1
AGWO	8.6	28.7	1
KGWO	7.9	26.3	0

## Data Availability

Data are contained within the article.
